# Anophtalmie bilatérale au cours du syndrome de Fraser: à propos d'un cas

**DOI:** 10.11604/pamj.2013.15.118.3037

**Published:** 2013-08-05

**Authors:** Zouheir Hafidi, Rajae Daoudi

**Affiliations:** 1 Université Mohammed V Souissi, service d'ophtalmologie A de l'hôpital des spécialités, Centre hospitalier universitaire, Rabat, Maroc

**Keywords:** Anophtalmie, syndrome polymalformatif, syndrome de Fraser, anophthalmia, Polymalformative syndrome, Fraser syndrome

## Image en médicine

Décrit pour la première fois en 1962, le syndrome de Fraser est un syndrome polymalformatif rare, transmis sur un mode autosomique récessif. Les critères diagnostiques sont cliniques, et comportent des critères majeurs: cryptophtalmie, syndactylie, anomalie génitale. Et des critères mineurs: anomalies des oreilles, du nez, du larynx et/ou du palais, les anomalies squelettiques, les hernies ombilicales, les agénésies rénales, et le retard mental chez les survivants. Pour poser le diagnostic, il faut réunir au moins deux critères majeurs et un critère mineur, ou bien un critère majeur et quatre critères mineurs. Nous présentons un nouveau cas de syndrome de Fraser retenu sur 2 critères majeurs et un critère mineur. Il s'agit d'un nourrisson de sexe féminin issu d'un mariage consanguin de 1er degré, amené par ses parents pour un syndrome malformatif facial et génital. A l'examen clinique on note un ankyloblépharon, avec une impossibilité d’écarter les paupières. La palpation trans-palpébrale ne révèle aucune structure individualisable sous-jacente. On note par ailleurs un élargissement de la base du nez. Le reste de l'examen général révèle une agénésie vulvaire, avec un orifice anal punctiforme d'implantation antérieure. Un bilan général a été réalisé fait d'un scanner cérébro-orbitaire révélant l'absence d'un bulbe oculaire au niveau de la cavité orbitaire. Le reste du bilan paraclinique est revenu négatif. Sur le plan ophtalmologique nous avons décidé une abstention thérapeutique, et le nourrisson a été adressé à un service de chirurgie infantile pour une prise en charge de l'anomalie anno-vulvaire.

**Figure 1:**
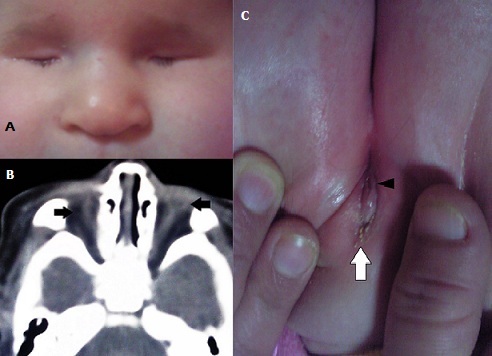
(A) : ankyloblépharon bilatéral avec affaissement palpébral en rapport avec l'absence du globe oculaire. (B) : absence de différenciation des petites lèvres et du clitoris (tête de flèche) avec une implantation antérieure de l'orifice anal qui semble être de petit diamètre (flèche). (C) : coupe scannographique cérébro-orbitaire confirmant l'absence des deux globes oculaires

